# The Transactions of the Provincial Medical and Surgical Association

**Published:** 1836-07-01

**Authors:** 


THE TRANSACTIONS OF THE PROVINCIAL MEDICAL AND SURGICAL ASSOCIATION. Volume IV. Octavo, pp. 578. London, 1836.

THE present volume of the Provincial Society's Transactions exceeds in bulk, if not in value, all its predecessors. It is, indeed, a formidable tome; and the reader of nearly six hundred pages must congratulate himself, if the quality is in any degree proportioned to the quantity.

The manner in which the work is constructed, renders it improbable that its circulation will ever extend materially beyond the circle of the members of the Association. A considerable portion of its matter is possessed of little more than local and temporary interest. There are many Addresses—proceedings and reports of councils and committees—elaborate topographical disquisitions, that few persons will be so patient as to read—and biographies of gentlemen, who, although amiable and excellent, have not had the good fortune to make the world inquisitive respecting either their names or their career. These are all matters which swell the volume—which are not, perhaps, out of place in it—but which, from the nature of things, must give it a local character and circulation.

Its arrangement and contents are the following:—

It consists of a sort of prologue, which ushers in six parts.

In the prologue, we are presented with Proceedings at the Anniversary Meeting at Oxford—Introductory Address, by Dr. Kidd—Report of the Council—Reports of Committees.

The first part contains an Address, delivered at the third Anniversary Meeting by Dr. Prichard.

In the second part is an Account of the Autumnal Fevers, Epidemic Scarlatina, and Small-pox, which prevailed at Amsterdam in 1834, by Dr. Nieuwenhuys.

The third part is topographical, and contains an Account of Malvern, by Mr. Addison, and of the Hundred of Penwith, by Dr. Forbes.

The fourth part is devoted to essays and cases. We have here a paper on the Physiology and Pathology of Intermittents, by Dr. Cowan of Bath—a Memoir on the Mode of securely closing moist Spirit Preparations, by our able friend Mr. Crosse, of Norwich—Observations on some of the injurious Effects of the Secale Cornutum, by Mr. Chavasse—a Case of Neuralgia of the Uterus, by Dr. Holbrooke—on the Unity of Organic Structure, by Dr. Dick—on Hydrometra, by Mr. Coley—a Case of Disease of the Female Pudendum, by Mr. Brayne—a Case in which the Cæsarian Operation was performed, by Mr. Knowles—Records of Ovarian Tumors, by Dr. Barlow—a Case of Fungoid Tumor, succeeded by Melanosis, by Dr. Norris.

The fifth part is dedicated to Statistics, and Reports of Hospitals and Infirmaries. In it, we find Statistical Observations on the Medical Charities of England and Ireland, by Dr. Walker—a Report of the Cases of the Out-patients of the Birmingham Infirmary—a Report from the Birmingham Eye Infirmary, and a Report from the Norfolk and Norwich Hospital. Finally, we have the Biography of the late Mr. Jennings, of Leamington, by Dr. Conolly.

Such are the contents of the volume before us. Were it not that many of the papers, and very much of the matter, are not adapted for notice here, and not likely to be read by any but those immediately concerned, we should turn with despair from the attempt at analysing six hundred pages. Fortunately, so few of them slip through the critical sieve, that our labour in collecting the grain below will be reduced to a moderate task. Our readers will thank us for dispensing with all notice of the Biography, Topography, and the Addresses.

The first article at which we shall glance is the Report of Dr. Nieuwenhuys. It is possessed of interest, and has been drawn up with care.

Art. I.—An Account of the Autumnal Fevers, Epidemic Scarlatina, and Small-pox, which prevailed at Amsterdam during the Year 1834. By *C. J. Nieuwenhuys*, M.D. &c.

The year 1834 would seem to have been a fatal one in Amsterdam. Intermittent fever, scarlet fever, and small-pox, conspired to destroy the Dutch in that city. That year was remarkable for its uncommonly mild Winter, cool Spring, and uninterruptedly hot Summer, succeeded by a pleasant and moderately fruitful Autumn, followed again by a mild Winter. As the Dutch know to a proverb that " hot Summers bring forth rich church-yards," even the very populace felt that mischief was brewing for them.

That mischief was considerable, for in Amsterdam, Hoorn, Eukhuizen, and Alkmaar, the excess of deaths over births varied from 1-5th to 1-10th. These places are situated on a marshy ground. In Rotterdam, Utrecht, St. Gravenhague, and Dortrecht, where the soil is solid or sandy, the births preponderated over the deaths, in the ratio of from 1-3d to 1-10th. A good instance of the influence of soil upon salubrity.

1. *Intermittent Fever.* It was in August that the intermittent fever began to assume a formidable aspect, and in October it reached its height. About the middle of December, the fever decidedly decreased, and catarrhal affections seemed to take its place. A more precise account of the mortality from fever is furnished by the following table. The accuracy of the classification of the varieties is admitted by the author to be questionable.

*Deaths from Fever in Amsterdam in* 1834.

| | Intermittents. | Bilious. | Catarrhal. | Total. |
|---|---|---|---|---|
| January | 29 | 1 | 12 | 42 |
| February | 12 | 0 | 14 | 26 |
| March | 33 | 1 | 13 | 47 |
| April | 25 | 4 | 19 | 48 |
| May | 30 | 2 | 15 | 47 |
| June | 30 | 3 | 15 | 48 |
| July | 23 | 3 | 5 | 31 |
| August | 37 | 12 | 20 | 69 |
| September | 74 | 49 | 18 | 141 |
| October | 125 | 66 | 25 | 216 |
| November | 145 | 37 | 29 | 211 |
| December | 103 | 5 | 16 | 124 |
| Total | 666 | 183 | 201 | 1050 |

The population of Amsterdam amounts to 202,364 persons, and the mortality from fever was, therefore, 1 in 192, or 1 in 8 of the whole deaths. Further, observes Dr. Nienwenhuys, if we take a *third* of the 556, who died of *dropsy*, and place them to the account of these fevers, they yield a total of 1235 ; but if to this number we also add *one-fourth* of the 1239, classed as dying under the head *exhaustion*, (and in each case we shall probably not be *beyond* the truth) we obtain a total of 1530 who died from these fevers or their results, or nearly 1 in every 133 of the whole population. Yet the mortality from these diseases was not so great as in 1826, when the same fevers raged through a vast population of Holland, commencing at Groningen. In that year, out of a population of 200,784 in Amsterdam, these fevers swept away 2390 individuals, being 1 in every 84. Conclusive evidence this of the too common insalubrity of Holland. The following is the Doctor's description of the disease.

"In the commencement, these fevers were generally of an *intermitting* character ; with some, however, they were *typhus remittens ;* in many cases medical advice was not sought until they had become *sub-continued ;* still an experienced eye could always recognise the *remittent* character. The *prodromi* in these fevers were, chilliness, which, sometimes trifling, was ascribed to the weather, listlessness, sickness with inclination to vomit, pain in the abdomen, and hypochondria; lastly, came the fever with coldness. It frequently came on also without any remarkable warnings, and then, in general, with violence. In children it was sometimes accompanied by fits; in persons of mature age, with violent symptoms, often with delirium. The tongue, at first slimy and whitish, became covered with a brown coat in the course of the fever, and at its acmé *black.* It was attended with violent pain at the *scrobiculus cordis,* oppression, particularly while the cold fit lasted, and extreme thirst. The motions were sometimes costive, sometimes relaxed; often there was a red sediment in the urine. If the progress of the disease was favourable, it mostly ended in a general perspiration, followed by lassitude, and, shortly after, by seeming tranquillity. It has been remarked, that the urine then discharged, left a white milky sediment, and that should the perspiration be but trifling, without this change in the urine, the fever remained obstinate, and the prognosis became unfavourable. I must acknowledge that I have not myself remarked these symptoms, but have observed a great tendency to debility, under every form of the disease ; and if the fever were not quickly suppressed, but from antiquated prejudices was treated, in consequence of the foul tongue and the gastric symptoms, by *anti-gastrica,* it was rendered worse, and often ended in death; even the *first* and *second* attacks were much increased, and accompanied with lethargic and nervous symptoms, which were considered as indicative of congestion on the brain. Notwithstanding some bilious symptoms, such as vomiting of bile and a yellow tint of the eyes and skin, were observable, particularly as the epidemic increased, (about August) yet I seldom witnessed them in so high a degree, as to warrant the epithet *bilious fever :* in fact, they were the *result,* not the *cause,* of the disease. Neither was inflammatory affection of the liver discernible, nor could I perceive that the spleen had been attacked. The fevers often declared themselves under different forms—sometimes as *febris larvata ;* I saw it commencing occasionally in children with convulsions ; in adults with apoplectic symptoms ; and, in some instances, it came on suddenly with delirium ; *twice* I saw it commence with vomiting of blood, and *once* with uterine hæmorrhage. If these dangerous symptoms were not relieved, or if medical assistance was not sought in proper time, the consequences were commonly fatal. I have seen a determination of blood to the head accompany this fever, evinced by redness of the face and a disposition to drowsiness, and by *delirium taciturnum,* but which symptoms vanished by aid of the specific used for the fever. Although I had no great reason to complain of *relapse*, yet examples of it were frequent, especially after an immoderate use of food, fatigue, and, above all, exposure to inclement or wet weather. Improper treatment or neglect was frequently followed by dropsy, affections of the abdominal viscera, and excessive exhaustion, which sent many to the grave." 68.

Such were the characters of the fever of 1834, the produce of the operation of a hot sun upon a marshy soil. Dr. Nieuwenhuys considers himself warranted in observing that if the disease was treated early the danger was not very great; but if allowed to become established, affections of the liver or spleen; chronic inflammation, or even abscess, of the intestines; dropsy; scurvy; and apoplexy, were the not unfrequent results.

The Doctor's treatment appears to have differed from that of many of his colleagues. It wears the aspect, philosophically speaking, of empiricism—that is, he gave one remedy, although the symptoms displayed great differences. When there was a foul and yellow tongue, violent oppression about the præcordia, and other indications of gastro-intestinal disturbance, many Dutch physicians prescribed emetics and purgatives. If, too, there were headache, flushed countenance, or lethargic tendency, they commenced with general or local bleeding before they exhibited quinine. But this, according to Dr. Nieuwenhuys, was a mistaken view, and he at once administered the specific medicine. Perhaps some of those excellent critics, whose experience enables them to pronounce on epidemics that they have not seen, and whose acquirements are such, that, although they may not have enjoyed many opportunities of practice, excepting those which the closet offers, they assure us that the profession is anxious to receive the law from their lips—perhaps, we say, these gentlemen may prove that Dr. Nieuwenhuys' notions are quite wrong, and that it is impossible he could have been successful. As the statement of that success, however, is only the expression of a fact, we must leave it as we find it.

2. *Scarlet Fever.* The annual average mortality from scarlet fever in Amsterdam, for forty years antecedent to 1834, was 72. The number of deaths in each year varied in a very marked degree, 18 persons only dying in one, and in another (1778) 369 having perished. Scarlet fever has been epidemic in Amsterdam in 1787, 1792, 1806, 1811, 1815, 1819, 1820. From 1821 to 1833, a period of 13 years, only 109 persons died of it.

But in 1834, it appeared with much virulence, and 498 individuals were carried off. It began as an epidemic in June, 1834. The number of deaths in each month of that year were—

| | | | |
|---|---|---|---|
| In January | 1 | In August | 50 |
| February | 0 | September | 78 |
| March | 0 | October | 136 |
| April | 1 | November | 106 |
| May | 4 | December | 61 |
| June | 22 | | |
| July | 39 | Total | 498 |

The epidemic began amongst the lower classes, and ultimately attacked the higher. Dr. Nieuwenhuys entertains the reasonable opinion, that although it was essentially epidemic in its origin and its diffusion, it might be, and it was, under favourable circumstances, contagious. He found, as with only a moderate scepticism he might have anticipated, belladonna of no use as a preventive.

" During the first months of its existence a subinflammatory complication was observable, but by the use of suitable remedies we succeeded tolerably well in subduing it. In addition to the usual sore throat, a *tracheitis* was sometimes remarked, which claimed much precaution, but after local bleeding it often happily subsided. In the latter months of 1834, a *nervous* form of the disease was undeniable, and demanded other treatment. In this form the throat was in a marked state of inflammation, and the membranes of the sensorium appeared to be much affected. Many sank under these symptoms, some so early as the third day, several before the eruption had diffused itself; in these cases it was only visible on the hands and neck. Bleeding, cold applications, &c. did not prevent this inflammation from terminating apparently in effusion; the sufferers nearly all died of this affection of the brain. I saw many cases of scarlatina, particularly in October and November, coexistent with *intermittent* fever, and in which, during the period of eruption, the fever decidedly intermitted, or, in some instances, became *sub-continued.* Fortunately, however, such forms of the disease were not general." 74.

In the majority of cases, the disease was either scarlatina simplex, or scarlatina anginosa, and did well under ordinary treatment. Dropsy was not an uncommon consequence, especially among the poor; and ulceration of the parotids, followed by deafness, was another sequela that was noticed. We see nothing to detain us in our author's account of the treatment he adopted. But the following extract is not uninteresting.

" It has been observed that some patients expired suddenly and unexpectedly during this epidemic, whilst, to the great astonishment of their relations, the physicians had sometimes held out but a few hours before, the most flattering hopes of recovery. In spite of every effort I was unable to discover the cause of this, until a similar misfortune occurred in my own practice, and which, I thought, accounted at least for some of these sudden deaths. Mr. C. a man 26 years of age and only six weeks married, had previously been tolerably healthy, but had complained of a pressure and contraction in the throat, resembling *globus hystericus,* and of a nervously sensitive constitution, was seized with sore throat on Wednesday evening, whilst supping with a friend. It continued the following day, on the evening of which I was called in, and found him in a high fever, which had come on with coldness. He was red all over. I prescribed a mixture *ex aq. sambuci, vino stabiato, acet. ammoniæ liquid., ex extr. cardui bened.* and a gargle of a decoction of figs. The next day, Friday, the fever was diminished, with continued eruption and extreme pain in the throat, which was red and swelled internally. To counteract this, I added a gargle *ex aq. rosarum et borace cum melle rosarum* to the foregoing medicines. In the evening the fever again exacerbated, coming on with coldness, increased sore throat, and continued *exanthema,* and lasting nearly the whole of the night. On the Saturday it went off as before, with perspiration. Two spoonfuls of a solution of quinine with *acidum muriaticum in aq. meliss.* was administered every two hours, and he seemed much better during the whole of the day; the *angina* was also so much better that he could be understood when he spoke. The same evening there was a slight exacerbation of fever, with increased sore throat and less redness. By continuing the remedies he became free from fever and sore throat on Sunday, and could swallow very well, but, according to his own description, was sensible of a strange contraction in the mouth, which hindered his speech. He said he felt otherwise well. However, the pulse was very quick and scarcely to be felt, which I attributed to extreme weakness after so violent an attack. As far as could be seen in the throat, it was covered with a cheesy matter, and the swelling had disappeared. He felt so much better, seemed to gain a little appetite, and, indeed, appeared so decidedly mending, that I shared the joy of the happy couple, little suspecting the snake in the grass. I continued the medicines as well as the gargle, although in a less quantity, but at my second visit on the afternoon of that day, how mournfully did I find myself deceived! He was perfectly sensible, but could not speak, only uttering a hoarse, incomprehensible sound, yet seemed contented, and inclined to say much. The pulse was no longer to be felt; the throat was blackish without swelling, and left no doubt of gangrene. At nine o'clock that evening he was no more." 78.

Another case of nearly a similar sort occurring, Dr. Nieuwenhuys, instructed by the former, adopted the treatment calculated, in his opinion, to arrest the sloughing. A patient who had bad sore-throat, without eruption, was all at once relieved of pain, but could neither swallow nor gargle, and could speak indistinctly only. The pulse was quick and low. The right tonsil was covered with a blackish matter, and the uvula was not visible. Dr. N. requested the surgeon in attendance to syringe the throat with a solution of creosote in rose-water (8 drops of the former to 6 ozs. of the latter), and to continue its use during the night; in addition to this, a spoonful of *infusum arnicæ cum æthere acetico et camphorâ* was administered every hour. Next morning, the patient could swallow and gargle. The tonsil was still discoloured, but no longer gangrenous. The creosote water was now made stronger, and used as a gargle. The patient recovered.

Those persons who were kept in over-heated rooms were especially affected, according to our author, with anasarca and scorbutic affections.

3. *Small-Pox.* In 1834, Amsterdam lost 134 persons with the small-pox. Some had been vaccinated formerly, apparently in an effectual manner. The following observations on this head are characterised by good common sense. He remarks that, if a comparison be drawn between the deaths by small-pox before the vaccine was introduced—during the time of its introduction—and after it was introduced, the balance in favour of vaccination will be proved to be considerable. By dividing, he says, 30 years into three eras, we see that during the first era, before the vaccine was introduced (1785, 1794), 6304 died of small-pox; in the second era, during its introduction (1795, 1804), 5446; in the third era after its introduction (1805, 1814), 2533.

It appears from this table, that as the cow-pox inoculation increased, fewer died of small-pox; for instance, during the last ten years, in which vaccination was very general, 3771 fewer persons fell a sacrifice to small-pox than in the first, when the art was unknown in this country; which places the usefulness of this salutary preventive beyond a doubt, and does away with the objections of every unprejudiced mind. Looking over the deaths in a later era of ten years, that from 1811 to 1820, they only amounted to 1498. This is the broad and correct view to be taken of the case. If vaccination has not brought perfect immunity from small-pox, it has diminished its mortality in a wonderful degree; and humanity is more directly beholden to Jenner than to any man, conqueror or sage, who has exercised his talents in its service.

II. Memoir upon the Method of securely closing Moist Anatomical Preparations, preserved in Spirit. By *John Green Crosse,* Esq. Surgeon to the Norfolk and Norwich Hospital, &c.

There are many things, which those who know them are apt to take for granted that other persons know, and which, consequently, are seldom generally explained or understood. The making of anatomical preparations is a piece of knowledge of this description. The curator of a museum, or an anatomical lecturer, has it at his fingers' ends, but the great majority of surgeons and physicians have no more notion of the proper way of making a spirit preparation, than they have of the square of the hypothenuse.

The consequence of this general ignorance is, that many valuable pathological specimens are not preserved at all, and that many more are preserved in a very imperfect manner. Our friends are constantly and kindly sending us preparations, which, from being improperly treated, are in most instances completely spoilt. This is a great pity, and affords an illustration of the observation that has been often made, that students neglect matters of daily occurrence, and, therefore, of great consequence, in their eagerness to witness extraordinary things, which probably will never materially affect them. A pupil would spend a whole day, or a whole week, in endeavouring to get a sight of the operation of removing the scrotum of a Hoo Loo, an operation which he never will have to perform, and which, if he did perform, would be of little service to his patient or himself—but he will not spend an hour in learning how to tie down a bottle in such a manner, that spirits of wine will not evaporate. This, however, is an age of utilitarianism, and our excellent friend Mr. Crosse, who has done much to improve the science of surgery, is employing himself in the useful and sensible task of informing his provincial brethren of the best mode of preserving preparations in spirit.

We will take the liberty of adding a few observations of our own, and as they are addressed to those who, we will suppose, know nothing of the matter, that is, to most students and many practitioners, we shall make no apology for their homely nature.

If the object is to preserve those colours in a preparation which washing would remove, it should immediately be placed in spirits of wine, and, after maceration for several days, it may be dissected, and suspended in the form which it is intended that it should permanently retain.

But if it is not necessary, as in the majority of cases it is not, to preserve those tints which depend on the presence of blood, or of matters that water will take up, then the morbid parts should be placed in water, and that should be changed twice or three times daily, until the blood is washed out. During this time, the preparation should be several times dissected *under water*, in order to remove the superfluous parts, and more particularly to get rid of the cellular and adipose tissue. When the blood is removed by maceration in water, the preparation should be transferred to a pan or stoppered bottle, containing spirits of wine. After maceration in this for a few days, it should be again carefully dissected *under spirit*, when the flocculent cellular membrane is removed, and the preparation properly *trimmed*. This is the part of the process which requires most care and judgment—if this is well done, the preparation will turn out a neat one, if it is ill-done, it will never look well. Several dissections are usually necessary, and the same spirit, being filtered through coarse white blotting-paper, will do again and again, both for the maceration and dissection.

The preparation is now reduced to its proper size and form. It is here that the taste, indeed the science of the dissector becomes apparent. It is then transferred to the bottle, and suspended in alcohol. We usually employ it of the strength of 56° above proof. This is expensive, but it is beautifully clear, and preparations both look and do better in it than in spirit of a weaker quality.

For small preparations, stoppered bottles are the best for this stage. For the preparation is not, in fact, *finally* put up. It ought to remain for some months in spirit. Stoppered bottles are, we say, best adapted for preparations of small size, for, in the first place, a good bottle permits very little evaporation, in the second, there is little trouble, and, in the third, there is no waste of bladder, lead, or paint. But stoppered bottles cannot be procured of sufficient size in the mouth to contain large preparations, and they must be placed in bottles secured in the manner we shall presently point out.

Whichever kind of bottle be employed, the preparation should be so adjusted, as not to interfere with the shape it is desired that it should ultimately assume. For it is so hardened by long immersion in the spirit, that if once it becomes corrugated, or assumes an improper form, it is in many instances impossible, and in all difficult, to restore the proper one.

In many preparations of hollow organs, it is desirable that the interior should be exposed, yet that the walls should not collapse. There are two modes of distending parts. If a canal, like the rectum, forms the preparation, it may be distended with baked horsehair or tow. But if it be wished to dilate a cavity with contracted apertures, as the heart, it should be injected with spirits of wine. The injection should be made under spirit, and very frequently repeated, in order that what escapes may be replaced, and that the organ should be kept as permanently distended as possible. In either case, whether the organ be distended with horsehair or with spirit injections, being immersed in spirit, it hardens, and, after a time, the parietes have become so rigid, that the cavity may be laid open without their collapsing. Some of the most beautiful preparations of sections of the bladder, heart, intestines, are thus made.

We have brought the preparation to a fit state for being permanently put up. We have not mentioned a variety of little circumstances, because they would detain both our readers and ourselves, and their own experience will instruct them better than many pages of minute and tiresome detail. We can now turn to Mr. Crosse, and follow him in his observations on the suspension of the preparation, mode of securing the bottle, and so on. We cannot analyse his recommendations, but must select those portions which are most distinct and forcible, and connect them by what slight critical cement may be required.

"I consider it injudicious," he says, "to attempt to close moist preparations in winter; the warmth of the succeeding summer will irresistibly expand the contained fluid, loosen and disturb the cover, and give rise to evaporation. The best, and at the same time most convenient, season for attending to this business, is during the warm and long days of summer; and where there is an option, the curator should make this choice. All the processes of closing a moist preparation are best conducted in warm weather; indeed, they can scarcely be accomplished quickly enough in the winter season. The jar being closed in warm weather, when the contained fluid has its greatest dimensions, is afterwards submitted to lower degrees of temperature only, which contract the fluid and do not, therefore, tend to displace the covering of the jar.

If we are too studious in avoiding expense, we cannot close moist preparations perfectly and successfully. The jars should be invariably made of the clearest flint glass, and should be from 1-10th to 1-8th, or even 1-6th of an inch thick. If the edge of a jar be not made sufficiently thick, it will break under the requisite force employed in closing the jar and adapting the lead to it. Oval jars seem to be required for some preparations, but should in general be rejected, on account of their being liable to crack in cold weather at the part where the curvature is greatest. Round glasses should be selected, shaped like *fig.* 1, *plate* 3; and it will be found very advantageous to have the jar high in proportion to its diameter, because, although some spirit should in the course of years evaporate, the morbid specimen may still remain covered with spirit, and the necessity of replenishing and re-covering the jar be obviated. There is also another advantage in a high jar, that it allows the upper part of the specimen to be distinctly seen. The lip of the jar needs to be very accurately formed; if the glass-blower have not performed his duty, leaving the lip of the jar irregular, or chipped, or not shaped exactly as I shall describe, no anatomist, in my opinion, can ever successfully close the jar. Suppose *fig.* 2, *plate* 3, to represent the perpendicular section of a jar, the top or rim should be exactly flat in the direction of the line *(a. b.)*, the lip *(b. c.)* should be rounded, and the rest of the surface of the glass should take the direction of the line *(c. d. e.)* If the rim be not well shaped, but irregular, and brought too near to *(e.)*, it will not be possible to apply the lead accurately, as will be subsequently described. Continually we find in use, jars with the rim bent irregularly, so that its inferior surface *(c. d.)* is found inaccessible when you are about to adjust the lead; and with such defects the jar can never be effectually closed." 291.

The preceding observations can be understood well enough without the diagrams, which we do not, therefore, introduce. We would insist very strongly on attention being paid to Mr. Crosse's hints with respect to the construction of the jars. We would add, that, for the beauty of the preparation, the glass should be extremely clear. Some glass has a sort of pearly tint; that spoils the appearance of the preparation. The majority of jars are made with an uneven bottom. We do not mean that the surface is so uneven as to give unsteadiness to the bottle, but that there is a sort of button left by the blowing, which refracts the light very unequally. This is obviated in bottles of the last and best construction, where the bottom of the jar is finished off very accurately, and no such refraction of the light occurs.

Mr. Crosse alludes to the preservation of specimens in spirits of turpentine, pyroligneous acid, solutions of certain salts, &c. All these plans are pretty generally discarded, and spirits of wine are now solely used in the best museums of the metropolis. We have stated that we employ exclusively alcohol, 56° above proof. Mr. Crosse remarks:—

" In general, the fluid employed for moist preparations, is composed of equal parts of spirits of wine and distilled water; and as these fluids do not at once mix completely together, the plan I advise, and have long practised, is, to put them together, and keep them for weeks or even months in narrow-mouthed glass stopper bottles, which are very high in proportion to their width; thus you get a perfectly clear and uniform fluid, which can be poured off as you require it, leaving the sediment at the bottom. If the water and spirits of wine be mixed and immediately used, the mixture has globules of air in it, as well as *impurities;* but *these* by long standing, in tall slender bottles such as I describe, will settle to the bottom, whilst the globules of air will soon arise to the top, leaving the proof spirit in the best state for being employed by the anatomist." 292.

The disadvantage of employing this comparatively weak spirit is, that sometimes the preparation becomes putrid in the centre. It never looks so well as the strong alkohol, but then the latter is exceedingly expensive. The retail price of alkohol, of the strength we employ, is twenty-two shillings and six-pence the gallon; and a gallon does not go far.

" Having selected," continues Mr. Crosse, "a jar suited to the size and shape of the specimen, you fill it nearly full with proof spirit, in which you immerse the specimen, transferring it direct from the glass-stopper jar, if no more dissecting be required.

The method of suspending a preparation must be left greatly to the ingenuity of the operator. Where a thread is employed, it should be very fine as well as strong; what is called dentist's silk answers well, a smaller or a larger filament being employed, according to the weight to be supported. For very large specimens, Mr. Macartney, when I was with him in Dublin, employed a cat-gut thread, such as is used by anglers to fasten the hook to the line; it is only specimens of great magnitude that require this. The thread being attached to the preparation, its two ends are carried in opposite directions over the rim of the jar; a fine thread is put circularly upon the neck of the jar; then the former two threads, held down by the circular one, are brought back over the rim of the jar, and meeting in the centre of its mouth, tied there." 298.

We would remark that the jar, before being filled with spirit, should always be rinsed out with spirit. It is usually rinsed out with water, but this is apt to render the liquid afterwards turbid. We would observe also that the preparation itself should be well washed with strong alkolol before it is transferred to the jar. This washing removes a number of flocculent debris, and it wastes no spirit, because the spirit that washed it is fit for the jar, when re-filtered through good blotting-paper. All these minute precautions make much difference in the appearance of the preparation, and we therefore mention them.

" In many instances a suspending thread may be dispensed with; where the specimen has been properly hardened and prepared, and the jar just fits it, you need only press it down to the situation you wish, and it will be retained there, compressed by the sides of the jar. Some specimens, particularly small ones, can be fixed upon talc, which rests at the bottom of the jar, and thus a suspending thread be avoided. Glass globes filled with air, as represented in *fig.* 3, *plate* 3, have been used, a thread attached to the specimen being hung upon the hook *(a)*: this method of suspending the preparation, by a floating glass globe, I have not found convenient; the preparation is unsteady, and sinks lower in the jar as the spirit evaporates." 293.

We would say, however, that in almost all cases it is better to employ a thread. Generally a preparation that rests upon the very bottom of the jar has an awkward look, and it seldom lies exactly even. In suspension the preservation of an exact level is indispensable for appearance' sake.

" The specimen should invariably be placed near to the bottom of the jar; often it may touch the bottom of the jar; and the upper third of the jar, or a considerable portion of it, should remain occupied only by the clear spirit; placing the specimen in this relative position to the top and bottom of the jar, not only brings the advantage already stated of allowing for the evaporation of spirit during many years, before the specimen will be exposed, and a necessity for renewal of the spirit arise, but admits light to come to the upper part of the specimen, enabling you to get a clear view of it, which is precluded when a preparation fills the jar near to the top, because the covering of the mouth of the jar is opaque." 293.

Mr. Crosse's objections to suspending the preparation high are very just. But, as we said before, we do not want to see it actually touch the bottom of the jar; at least, not in ordinary cases. We now come to a very important part of the process.

"The preparation being suspended, or supported, or somehow fixed in the right relative position in the jar, you must proceed to fill the jar most accurately to the brim with proof spirit, which should be poured out of a small tall and narrow vial (*fig.* 5, *plate* 2); for this filling to the brim cannot be done with accuracy, if you pour the proof spirit out of a large bottle, nor unless you make the brim of the jar exactly horizontal.

You next proceed to close the top of the jar, and bladder is the first covering you put over it. A common bullock's bladder, inflated and dried as we commonly purchase it, is the best material; and you must immerse it in water, and keep it macerating in this fluid, until it is pulpy, or until one coat passes over the other readily when a portion of the bladder is rubbed between the finger and thumb. A week or ten days will be required for macerating a bladder well, even in the warmest weather; and in the cold season two or three weeks. In an emergency you may expedite the process by using tepid water; it can scarcely be macerated too much, though nearly putrefying, provided it continues sufficiently firm not to tear under the force requisite in applying it. The bladder thus macerated should be well washed with water to render it quite clean, and wiped with a towel to remove moisture from its surfaces. Having also wiped the rim of the jar dry, you proceed to apply the bladder, by placing the inner or mucous surface next the spirit, laying it over the top of the jar and in contact with the spirit, so that no air is left; you bring the edge of the bladder round the rim of the jar (*b. c. d. fig.* 2, *plate* 3), and maintain it in this position by several turns of twine round the neck of the jar. Cut off the bladder close to the twine all round the neck of the jar, and press the edge of the bladder firmly upon the neck of the jar, by wiping it from above downwards with a towel, that on drying it may adhere to the jar firmly, and by a thin edge.

Experience has suggested to me a plan of drying the bladder, which is of great utility: that part only should be dried which is in contact with the rim and neck of the jar, and not the central portion which is over the mouth of the jar and in contact with the spirit. If no precaution be taken, whilst you are allowin the first bladder to dry, much spirit will evaporate through the portion of the bladder covering the mouth of the jar. I therefore cover all the bladder which is over the mouth of the jar and in contact with the spirit with a circular flat piece of lead, of such size as answers to the mouth of the jar. Graduated sizes of these circular pieces of lead should be kept (as *figs.* 4, 5, 6, *plate* 3), which may be an eighth of an inch or more in thickness; the weight of the lead brings some advantage; this lead should be allowed to remain on until the bladder is perfectly dried upon, and firmly adherent to, the rim of the jar. The bladder being perfectly dry upon the rim and neck of the jar, you remove the circular lead from the top, and also the twine placed round the neck of the jar; then pare off any irregularities of the edge of the adherent bladder at the neck of the jar, by cutting obliquely from above downwards." 295.

We are in the habit, as the curators of most museums are, of introducing talc between the bladder and the spirit. There are several advantages attendant on the employment of talc. In the first place, it affords a great convenience for the suspension of the preparation. Instead of carrying the suspending string over the rim of the jar, and securing it round the neck by the circular thread, it is carried more perpendicularly through a small hole drilled in the talc itself. In this way it hangs better in the bottle, and being fastened to the talc, it can be removed and returned into the bottle again, or into another with facility. In the second place, the talc prevents the bladder from becoming concave, a very unsightly occurrence when it takes place. And, in the third place, the talc forms the best preservative of all against evaporation from the superficies. The talc, itself, should be cut into a circular piece, not extending quite so far as the circumference of the bottle's rim. We are sure that if Mr. Crosse tries the talc fairly, he will admit its utility and excellence. The talc should be laid on the surface of the spirit, and over it the first bladder should be applied in the manner recommended by Mr. Crosse.

The drying of the first bladder in warm weather will occupy at least twenty-four hours, and sometimes as much as three or four days. To expedite it the jar should be placed where there is a free current of air.

The next step is to apply a thin sheet of lead over the bladder. The lead is very instrumental in obviating evaporation, and its proper adjustment is therefore a matter of much delicacy and importance. The sheet lead should be thinner in proportion as the jar is smaller. For small jars, an inch or two in diameter, Mr. Crosse is inclined to think that the lead should be the 1-100th of an inch in thickness—for the largest jar, the 1-40th of an inch, or about the thickness of a common wafer. The following is Mr. Crosse's description of the method of applying it.

"You cut out a piece of the prepared lead, as much larger than the rim of the jar as is required to lap over to the extent I shall describe, and having rendered this lead perfectly smooth and regular, you lay it upon the top of the jar, and with the fingers, aided by instruments of wood or ivory, such as are represented in *plate* 4, *figs.* 1, 2, 3, you put the lead in contact with the first bladder, and by forcible pressure with one or other of these instruments, you mould the lead exactly to the shape of the rim and neck of the jar, after the manner that a shoe-maker uses similar instruments in pressing down and smoothing the edge of the sole of a shoe. The object is to have the lead correspond to as much of the rim of the jar as is possible (the bladder, of course, always intervening), and with this view, you make the portion of the lead, answering to the mouth of the jar, a little concave, taking the same shape as the bladder has assumed in drying; you adjust the lead first to the inner edge of the rim (at a *fig.* 2 of *plate* 3), then to all the upper flat surface of the rim (*a. b.*), then round its outer edge (*b. c.*), to the under surface of the rim (*c. d.*), and downwards upon the neck of the jar to (*e.*). The leading of a jar, after this fashion, is necessarily a slow process; but as every thing mainly depends upon it, no trouble should be spared. When you have gotten the lead tolerably applied, being every where in contact with the first bladder, you must, by pressure and hard rubbing with one of the wooden or ivory instruments (*plate* 4, *figs.* 1, 2, 3), put it firmer down upon the bladder and rim of the jar, using about as much force as the rim of the glass will bear, and adopting the same motion of the instrument as a gilder in burnishing a frame. The lead being everywhere smoothly and firmly applied, you finish by paring off the lead all around the neck of the jar, cutting obliquely from above downwards, and leaving the lead long enough to pass a little lower upon the neck of the jar than the first bladder." 299.

The representations of the instruments employed for moulding the lead are of no great consequence, as they are merely flat or oval pieces of ivory or hard wood, set in a convenient handle.

Over the lead comes the second bladder, which should be well macerated, &c. as before. In applying this bladder to the lead, proceeds Mr. Crosse, you may put the inner or mucous coat outwards (the reverse of the first bladder), and the bladder should everywhere touch the lead, without any intervening air, being secured by several turns of twine round the neck of the jar. After the twine is applied, and tied firm, you cut off the bladder even and regular just below it, taking care to leave enough of the bladder to cover the edge of the lead, and descend just below it. In order to make the edge of the bladder stick well to the neck of the jar, you must press it down firmly with a rough towel, or with the handle of a knife, if the bladder have been well macerated, it will stick very close in drying, but a half-macerated bladder will turn up at the edge and not adhere. Having finished the second covering of bladder, you leave it to dry for twenty-four or forty-eight hours, and when dry, you make its edge perfectly regular upon the neck of the jar, paring off all irregularities with a sharp knife, in the same direction as in paring the lead or first bladder.

When the second bladder is perfectly dry, it should be painted over with common black paint, which is to extend beyond it on the neck of the jar. When this coat of paint is dry, it should receive a wash of coach-maker's black varnish. Once in a few years a fresh coat of black varnish will be required, but, with this exception, Mr. Crosse assures us that the preparation will require little or nothing to be done to it for thirty, or even fifty years. We must say that we have not been so fortunate in the preparations we have put up, but no doubt we were not so exact in some parts of the process as we should have been.

Mr. Crosse very properly remarks, that a knowledge of the mode of putting up preparations is almost as necessary to the attainment of much pathological knowledge, as the dissection of the body itself. It cannot then be beneath the attention of every one who is anxious to become acquainted with morbid anatomy. Such information is absolutely indispensable to all who aspire to practise their profession with credit. The same view of the practical utility of preparation-making, which influenced Mr. Crosse in publishing his observations, has influenced us in giving them extended currency, and in adding a few remarks of our own. The station which Mr. Crosse himself occupies, is the best proof of the eminence and the respect, to which a zealous cultivation of both the scientific and practical department of the profession leads.

Before we quit this subject, we would offer another observation. It is often advantageous to preserve a dissection in spirit for use. We wish to take it out occasionally, and to examine it. We are not solicitous about it as a preparation, but we merely desire to preserve the dissection in a serviceable condition, at as small an expense as possible. This may be done with the *vapour* of alkohol. Let the preparation be placed in a jar, and alkohol poured on it to the level of half an inch from the *bottom* of the vessel. This is to be tied over with bladder and leaded, and the vapour of the spirit will preserve the parts in a perfect state. When it has evaporated, it must, of course, be renewed. This is a very economical method of preserving fine dissections for lecture, and it is much employed in the great schools of London.

III. A Case of Deformity of the Pelvis, in which the Cæsarean Operation was performed with Success. By *S. B. Knowles*, Esq. Lecturer on Botany at the Birmingham School of Medicine.

This is a very interesting case, and does, we think, much credit to all the parties concerned.

*Case.* Sarah Bate, æt. 36, was delivered of her first child in May, 1824—of her second in December, 1826. Both are now living, but, without any appreciable disease of the spine, have lost the use of their lower extremities. In May, 1828, she had a girl still-born, and in Nov. 1829, a boy born prematurely. During her pregnancy with this child, she began to perceive a loss of power in her lower extremities, with considerable uneasiness, though not amounting to severe pain, in her hips and loins. Soon after her delivery, which was in the seventh month of utero-gestation, she again became pregnant, when the loss of power increased to such a degree, that she became incapable of moving from her seat without the aid of crutches. After this period she miscarried four times, and at rather short intervals.

In her last pregnancy, the ninth, and that in which the circumstances we are about to mention occurred, she went her full time. Mr. Knowles was summoned to her at 7, a.m. of the 15th May, 1835, labour having commenced about 4, p.m. of the 14th. The pains were neither strong nor frequent. The legs and thighs were extremely anasarcous. Mr. K. left her, but was again summoned at 3, p.m. when he found her in strong labour, the pains being vigorous and frequent.

Upon examination, he observes, a most extraordinary state of deformity presented itself; the sacrum, with the lower lumbar vertebræ projecting, and at the same time descending so far into the cavity of the pelvis, as to occupy a large portion of its space. This projecting body was also rounded in shape in a most remarkable manner; so that without a somewhat careful examination, it might easily have been mistaken for the child's head. Upon passing the finger, however, between it and the hollow of the sacrum, a perfect continuity of surface might be traced from one to the other, thus shewing at once the actual nature of the case. The capacity of the pelvis was still further diminished by the ossa pubis, which were bent inwards to such a degree, as to approach very nearly to the distorted vertebræ. Of course it was impossible to ascertain the state of the presentation.

At 4, p.m. a violent pain ruptured the membranes, and an unusually large quantity of the liquor amnii escaped; but it was still impossible to feel the child.

"Under these circumstances, I requested the assistance of my friend Mr. Ingleby, the able and intelligent Lecturer on Midwifery at the Birmingham School of Medicine. After the most careful examination, we ascertained that the space left by the deformity did not exceed two inches in length, by less than one in breadth; a space that could by no means be considered available under any point of view. To attempt to open the head, where that head could not be felt, would have been neither prudent nor practicable. Admitting, however, for a moment, the bare possibility of opening the head under such circumstances, still the subsequent extraction of the child must have been totally impracticable. It was evident, therefore, that the only course which afforded a prospect of saving either the mother or the child, was the performance of the Cæsarean operation." 378.

The case was explained to the patient, who immediately gave her assent to the operation. Mr. Wood, senior surgeon to the Birmingham Hospital, Mr. Wickenden, and Mr. Heath, were joined in consultation, and, with their assistance, the operation was performed by Mr. Knowles at 11, p.m. thirty hours after the commencement of labour.

"The external incision, which was made through the linea alba, passed immediately on the left of the umbilicus, and was about ten inches in length. A portion of intestine immediately protruded, which was carefully kept back by an assistant. The incision into the uterus was commenced near its fundus, by passing the scalpel carefully through its parietes (which were about an inch in thickness), so as to allow the admission of two fingers. Using my fingers as a director, I rapidly enlarged the opening, by which the placenta was exposed, accompanied by a brisk hemorrhage. Passing my hand by the side of the placenta, I ruptured the membranes, and without difficulty took out the child, which was living. The placenta was then detached, some coagula were removed, the uterus contracted, and the hæmorrhage (which probably did not exceed sixteen ounces) was immediately arrested. The external integuments were brought together by stitches, which were inserted at somewhat more than an inch apart; adhesive straps were applied in the intervals, and the whole was secured by a compress and bandage." 380.

The patient bore the operation without a murmur. In an hour after it she took an opiate, and at 3, A. M. of the 16th she fell asleep. In the morning she had some vaginal discharge, and a good deal of fluid escaped from the lower part of the wound. Having voided no urine, a catheter was introduced, after which she passed considerable quantities. Next morning she complained much of flatulence, and vomited; pulse 110, and full. An injection of mutton-broth was administered, and after it one of turpentine, but little save flatus was brought away.

" 18th, eleven, a. m. Has had considerable pain, with vomiting, during the night, but has slept at intervals : the tympanitis has increased. She was now ordered to take, at intervals of two or three hours, equal portions of soda water and milk, with, occasionally, a little chicken broth. The wound was dressed this morning for the first time ; it looks healthy, but no union has taken place. There is in fact, so much distention, that it is scarcely possible to keep the lips of the wound in apposition ; the stitches are kept upon the stretch, and a portion of intestine is visible through one of the intervals. Four, p. m.—She is become thirsty, and has distressing hiccough; there is also a general tenderness of the abdomen, with constant, though not violent pain. The mixture has acted gently on the bowels. Ordered a draught, containing tinct. opii, ♏xxv. to be taken immediately. Nine, p. m.—Thinks herself relieved by the draught; the hiccough continues but is less violent. Ordered a draught with tr. opii ♏l., to be taken at bed-time." 381.

She passed a better night, and next day all the symptoms were rather mitigated. The wound looked well, with the exception of two or three small sloughs, in connexion with the stitches. There was still some lochial discharge from the vagina, and a small secretion of milk. To produce contraction of the uterus, the child was applied to the breast occasionally. Pulse 145, tongue clean, though somewhat dry. She passed much flatus per anum, which relieved the tympanitis.

We see no necessity for pursuing the subsequent details. The wound did not unite by the first intention, but gave issue to a serious discharge, succeeded on the tenth day after the operation by one of a purulent character. Granulations then formed, and in about a month the wound was filled up with them. The treatment consisted in opiates—occasional injections—soda water and milk, gruel, and chicken broth, succeeded by the stronger broths, porter, and light tonics. The sensible view which Mr. Knowles took of the case was this—that the woman was in a very debilitated condition, and that her best chance, on the whole, would arise from the total absence of depletion. He thinks too, and in such a case the supposition is probably correct, that the non-adhesion of the wound was rather favourable than otherwise, by permitting the free escape of fluids.

*(To be continued in our next Number.)*

